# Supplementary material for: Zika virus-like particle vaccine protects AG129 mice and rhesus macaques against Zika virus
Source: PLoS Negl Trop Dis. 2021 Mar 12;15(3):e0009195. doi: 10.1371/journal.pntd.0009195 (PMC7990201; doi:10.1371/journal.pntd.0009195)
Supplement: Attachment — Submitted filename: PNTD-D-20-01817 Review.docx [file pntd.0009195.s002.docx]

**PNTD-D-20-01817:**

**Zika virus-like particle vaccine protects AG129 mice and rhesus macaques against Zika virus**

Authors have tested immunogenicity and efficacy of a previously developed ZIKV prM/E VLP vaccine in A129 mice and NHP primates. Authors claim that co-administration of alum as an adjuvant with their VLP vaccine is dose-sparing. The manuscript could benefit from more proofreading (for grammatical mistakes and English editing) and to improve the flow. It was very hard to follow the results section, as data was not written properly in the text nor were the methods.

In the introduction it would be important to mention other ZIKV vaccines that are in development especially other VLP vaccines and explain the novelty of their approach. A link to and more background on their previous work (study by Espinosa et al 2018; https://www.sciencedirect.com/science/article/pii/S2352396417304917#s0045 ) needs to be provided. This will help explain the background of this VLP candidate and point out the novelty and significance of the current (presented) work. It is also important the authors point out that this VLP is a chimera of 2 ZIKV strains. It would be appreciated if the authors can specify that in the introduction and also specify the details of the ZIKV cassette plasmid in methods (line 117).

It is necessary that the authors explain the novelty of their vaccine candidate – ie how does it compare to other ZIKV VLP vaccines and envelope-based vaccines? Discussion needs more detail (depth), not just sweeping statements on 3 vaccine candidates and alum as an adjuvant.

Data on the VLP production and purification (Figure 1) is not novel, as it was reported in the authors’ previous publication by Espinosa et al 2018. Also, as there is no mention of E stem anchor in the plasmid design (line 117- VLP design, purification and characterisation) nor is it indicated on the plasmid schematic- please correct this and explain. Maybe this Figure should be a Supplementary figure outlining vaccine design and manufacture as it is a repetition of what has been previously published (the wording in Fig legend is exactly the same as what has been published previously in Espinosa et al 2018).

Also, it would be nice to have the VLP formation confirmed by TEM. The authors do not necessarily need to do this for this publication as they have shown previously that VLPs are made- however it would be good if they can provide assurance that they perform batch testing of their VLP preparations.

**Immunogenicity in AG129 mice**

Correct the subheading line 360- it is AG129 mice not AG19. Link data that is being discussed to figures at end of sentences.

Data in Figure 2B is not discussed, there needs to be a better link between nAb levels after prime and after boost (Fig2C). Has statistics been done to compare the different doses, as most of the reported statistics are when compared to alum only group? Please specify if so. You state that all but some vaccinated groups have significant levels of nAb when compared to control, but you have obviously done a titration study to determine the optimal and/or minimal dose required for protection. As such you need to show if there are any statistical differences between different doses.

What was the correct challenge dose? In methods (line 157) authors specify that mice were challenged with 100-200 PFU of PRVABC59 but in the figure legend of figure 3 they say ~100CCID50 (line 400)?

**NHP immunogenicity and efficacy**

How did the authors predetermine a 15:1 alum:VLP ratio for NHP immunisation dose?

Fig 6A- I am confused by multiple blood draws after challenge- there appear to have been 8 blood draws in a time period of 10 days

Line 465- specify when the blood samples drawn

Line 467: on which days was humoral immunity evaluated? If at every blood draw, say so?

Line 468: on what days post challenge was VL quantified?

Figure 6: figure legend. I am completely confused by animal numbers? Here you say 6 NHP per group, in methods you say 5 per group with an extra one added randomly. Where? Your graphs clearly show data for only 5 animals per vaccine dose? Please be specific for each group and correct text accordingly.

Again, please if you are doing a dose titration study you must specify results obtained for each. You also must compare different doses and note if significant or not (for nAb data).

Authors also show nAb titers 15 days post-challenge (day 70/71) but do not mention them in results section. Please correct this. Either remove them from the graphs or report the data.

For the NHP model, it would be helpful to say what viremia looks like when NHPs are challenged with this dose, i.e. when does it peak and when is it cleared?

Line 491- if 20ug dose was not significantly greater than 5ug dose, specify. Also specify if 5ug was greater than 1.25ug dose?

Line 577: authors mention that a challenge dose may account for the failure to detect infection in alum only control NHP. I disagree with this statement as many different studies have shown that rhesus macaques can be infected with lower doses than what the authors have used in their study (some have even used 10^3^ PFU (Abbink et al 2017, Sc Transl Med)).

Line 582: There are many more vaccine candidates that have been developed using ZIKV structural genes, not just the 3 that the authors mention. They should be mentioned (vectored, inactivated ZIKV….).

Minor comments:

Line 42: As ZIKV infection has been causally linked to CZS, reword the sentence (remove word potentially).

Line 123: Please provide details on VLP purification or if the same as in Espinosa et al 2018 link to the reference.

Line 186-Please specify what the vaccine dose/alum groups were and how many animals per group? i.e. 20ugVLP/300 ug alum – (2 males, 3 females) and so on.

Line 364, for clarity can you please specify the challenge dose and route

Line 366 “induced upon a prime “ remove “a”

Line 367 “following the a second” remove “a”

Line 399: correct Study day 44

Line 387 Please specify vaccine doses and also specify the challenge dose and route.

Line 465: correct PRVABC59 and specify dose and route of challenge

Line 540: please specify statistical test that was used
