# Supplementary material for: Zika virus-like particle vaccine protects AG129 mice and rhesus macaques against Zika virus
Source: PLoS Negl Trop Dis. 2021 Mar 12;15(3):e0009195. doi: 10.1371/journal.pntd.0009195 (PMC7990201; doi:10.1371/journal.pntd.0009195)
Supplement: Attachment — Submitted filename: Response to Reviewers Comments 29Dec2020.docx [file pntd.0009195.s003.docx]

**Reviewer's Responses to Questions**

**PNTD-D-20-01817:**

**Zika virus-like particle vaccine protects AG129 mice and rhesus macaques against Zika virus**

<b>Key Review Criteria Required for Acceptance?</b></br></br> As you describe the new analyses required for acceptance, please consider the following:</br></br>

<b>Methods</b></br></br>

-Are the objectives of the study clearly articulated with a clear testable hypothesis stated?</br> -Is the study design appropriate to address the stated objectives?</br> -Is the population clearly described and appropriate for the hypothesis being tested?</br> -Is the sample size sufficient to ensure adequate power to address the hypothesis being tested?</br> -Were correct statistical analysis used to support conclusions?</br> -Are there concerns about ethical or regulatory requirements being met?</br></br>

Reviewer #1: The manuscript describes the efficacy of a Zika virus (ZIKV) virus-like particle (VLP) vaccine in AG129 mice and non-human primate. The authors follow the generation of neutralizing antibody using multiple assays and RNAemia following challenge. The methods outlined in the manuscript are acceptable and clearly defined.

Minor comments-

1.	Please define the contents of the “formulation buffer” on line 125 (Line **144** Tracked Changes document). The following buffer contents were noted (Line 144 Tracked changes) 25mM TRIS hydrochloride and 25mM sodium citrate dihydrate

2.	Please provide an explanation for using different alum doses for the non-human primate studies, since the dose of alum remained consistent for each VLP dose in the mouse studies.

(Line **230** Tracked Changes document)

We decided to titrate both the VLP and alum doses in the NHP studies in contrast to the mouse studies that held the alum dose constant for each of the VLP titration doses. This was done to increase the likelihood of observing a vaccine ‘dose-effect’ on immunogenicity and efficacy by reducing both VLP and alum dosage. This language is now included in the Materials and Methods section (Line **230** Tracked changes) to inform the reader why this dosing strategy was used.

Reviewer #2: The Methods section is cluttered and includes redundancies, such as the information about the macaque cohort. Redundant information in the ethics statement for rhesus macaques that was also addressed in the Immunogenicity and efficacy studies section was removed. Specifically, (**Line 124 Tracked changes** **document**) ~~A total of 22 (11male and 11 female)~~ ~~Chinese-origin rhesus macaques (~~*~~Macaca mulatta~~*~~), referred to as nonhuman primates (NHPs), weighing 3.8 – 6.3 kg and being 3.1 – 5.7 years of age at initial dosing were procured from Covance Research Products (Alice, TX)~~.

The use of multiple methods to measure neutralization is impressive.

Reviewer #3: Yes. But methods section needs to be written more concisely and with more clarity. Furthermore statistical analysis of different sample groups need to be done, not just compared to mock (alum groups).

The Materials and methods section has been modified to include information to increase clarity as requested in specific reviewer comments/requests. In regard to statistical analysis of different sample dose groups, these were generally not significant. However, outcomes of these analyses are now included in the text. Results have been revised to indicate the absence of significant differences between VLP groups for every statistical test performed. In many cases, when pairwise comparisons were not significant, ‘ns’ was not included in the Fig due to not wanting to clutter Fig and make unreadable.

Examples indicating statistical analysis was performed on all pairwise comparisons

(Line **349** Tracked changes document), “Serum nAb titers generated by PRNT_50_, MN_50_, and RlucNT_50_ assays; infectious virus titers by plaque assay; and RNAemia levels by RT-qPCR (Log Relative GE for mice and GE/mL for NHP) were compared within each time point by Kruskal-Wallis nonparametric ANOVA followed by Dunn’s comparison tests between all pairwise comparisons. “

(Line **418** Tracked changes document), “Kruskal-Wallis analysis followed by Dunn’s comparison tests on all pairwise comparisons within each time point were performed and statistical significance levels relative to each alum only group are denoted above the GMT value as: ****P < 0.0001, ***P < 0.001, **P < 0.01, *P < 0.05, or ns (not significant). Additional significant differences between pairwise VLP groups are shown by bars and significance levels. “

(Line **421** in Fig 2 Legend, Tracked Changes document) “Additional significant differences between pairwise VLP groups are shown by bars and significance levels.”

(Line **431** in Tracked Changes document), “. In addition, the 0.1µg VLP group survival was significantly lower than that of the 3 fully protected groups (10µg VLP + Alum, 1µg VLP + Alum, and 10µg VLP, P = 0.033 Two-tailed Fishers Exact Test), but no other statistically significant differences were achieved.

(Line **459** Tracked changes document), “. There were no statistically significant differences in the GE levels between VLP groups. “

(Line **473** Tracked changes document), in regard to RNAemia “All possible pairwise comparisons were performed and there were no statistically significant differences among VLP groups. “

(Starting line **534** Tracked changes document), “ significance between groups shown by lines and significance levels by Kruskal-Wallis and Dunn’s pairwise comparison tests denoted by: ****P < 0.0001, ***P < 0.001, **P < 0.01, *P < 0.05, or ns (not significant).

(Line **543** Tracked changes document), in regard to NHP nAbs, day 28 “There were no significant differences in nAb responses between the different VLP dose groups.

(Line **545** Tracked changes document), in regard to NHP nAbs, day 56 “However, there were no significant differences in nAb responses between the different VLP dose groups.

(Line **583** Tracked changes document), “No statistically significant differences in viral load on any day were observed (Kruskal-Wallis plus all possible Dunn’s pairwise comparison tests)

--------------------

<b>Results</b></br></br>

-Does the analysis presented match the analysis plan?</br> -Are the results clearly and completely presented?</br> -Are the figures (Tables, Images) of sufficient quality for clarity?</br></br>

Reviewer #1: The results are easy to follow and match the analysis. The figures are clearly presented.

Reviewer #2: Minor modifications requested:

Does the isolate of PRVABC59 contain the variants known to be associated with attenuation (Duggal et al, <https://doi.org/10.1016/j.virol.2019.02.004>)? if so, the authors should cite this paper and state the caveats associated with this virus. The data are still valid as this isolate has been used extensively but this information is important for interpretation.

(Line **651** Tracked changes document)

We thank the reviewer for noting the Duggal ref. Both PRBABC59 viral stocks used in the mouse and NHP studies were obtained from BEI and passaged. It is unknown whether the attenuating mutations variants at positions E-V330L and NS1-W98G were present in the challenge virus for the mouse studies. However, the virus was sequenced prior to the NHP study. We have now cited this reference and noted the caveats associated with the virus. The text in the Discussion now reads, “It should be noted that low titer passage of PRVABC59 has been shown to acquire mutations in E (V333L) and NS1 (W98G) which attenuates pathogenesis in mice [Duggal Virol 2019]. It is not known how these mutations affect viral pathogenesis in NHPs. The virus was sequenced prior to the NHP challenge whereby the consensus sequence (more than 50% of the sequences assessed) did not have the E (V333L) mutation but did have the NS1 (W98G) mutation in approximately 62% of the sequences tested. This mutation may or may not be a contributing factor in low level of viremia detected in the NHPs.”

- some figures represent data post-vaccination but are focused on data post challenge. this is fine, but could be clarified by, for instance, showing the number of days post challenge in parentheses. This is clear in the text, but the figures as they stand are a bit confusing.

To clarify understanding of the timing, **post-challenge Figs** and text were converted to post-challenge rather than study day, or in some instances, both are given. Days post-challenge are now indicated for Figs: 4, 5, 8, and 9.

**Reviewer #3: yes. see attached file for detailed comments**.

--------------------

<b>Conclusions</b></br></br>

-

Are the conclusions supported by the data presented?</br> -Are the limitations of analysis clearly described?</br> -Do the authors discuss how these data can be helpful to advance our understanding of the topic under study?</br> -Is public health relevance addressed?</br></br>

Reviewer #1: The data presented supports the conclusions of the manuscript.

Reviewer #2: -The authors state the dose used in the macaque studies (~10,000pfu) could be too low or the species not ideal resulting in inconsistent detection of infectious virus. The discussion would benefit from comparison with published studies as various viral inoculum doses and macaque species (e.g. cynomolgus, pigtail) have been used.

Reviewer #3: yes

(Line **660** Tracked changes document)

There seems to be some confusion regarding this topic. We made the statement that the dose of the challenge virus may be too low for detection of viremia, i.e., live virus detected by a plague assay. We and other groups have detected RNAemia by RT-qPCR, a more sensitive assay, typically in the range of 1 x 10^4-7^ RNA copies/mL. We have added the following language to clarify, “Other groups typically use RNAemia as measured by RT-qPCR as a readout of efficacy. Viral challenge doses as low as 1 x 10^3^ PFU or FFU achieved levels 1 x 10^4-7^ copies of RNA/mL which is comparable to levels noted in our studies [Dowd Science 2016; Abbink Sci Transl Med 2017]. Cynomolgus monkeys have also been used as a model for RNAemia demonstrating again comparable levels of RNA copies in the blood following viral challenge in the range of 1 x 10^4-5^ [Koide Front Microbiol 2016; Osuna Nat Med 2016].”

--------------------

<b>Editorial and Data Presentation Modifications?</b></br><br/> Use this section for editorial suggestions as well as relatively minor modifications of existing data that would enhance clarity. If the only modifications needed are minor and/or editorial, you may wish to recommend “Minor Revision” or “Accept”.

Reviewer #1: Minor comments-

1.	In all the figures related to the mouse studies, the 0.1ug VLP + alum group is missing the word “VLP”.

The Figs have been modified to add ‘VLP’ to the 0.1µg VLP + alum groups, i.e., Fig 2B, Fig 2C, Fig 3B, Fig 4A.

Reviewer #2: (No Response)

Reviewer #3: (No Response)

--------------------

<b>Summary and General Comments</b></br></br> Use this section to provide overall comments, discuss strengths/weaknesses of the study, novelty, significance, general execution and scholarship. You may also include additional comments for the author, including concerns about dual publication, research ethics, or publication ethics. If requesting major revision, please articulate the new experiments that are needed.

Reviewer #1: The study is well written and thought out. While other VLP based vaccines against ZIKV have been tested before in mice, this seems to be the first report in non-human primates. The authors missed an opportunity to show that the antibodies generated following vaccination with this VLP scheme are unlikely to promote antibody-dependent enhancement (ADE), which will be important prior to moving into human trials. Overall, the data supports the conclusions of the manuscript.

We thank the reviewer for their comments on whether this VLP has potential to promote ADE. We feel this is beyond the scope of this manuscript. However, we are well aware of the necessity to evaluate ADE.

General Comments and Experimental suggestions-

1. Are the structural proteins on the virus-like particles produced fully mature or are there a mixture of mature and immature particles? Please discuss.

The reviewer correctly pointed out that we did not indicate/emphasize the level of maturity of our ZIKV particle. This was not a focus of the study and we do not have data to address this issue. However, we agree with the reviewer that it is a topic to evaluate further.

2.	The vaccination schemes generated robust neutralizing antibody responses. It would be interesting to know if the majority of the antibodies generated were type specific and/or bound to domain III of the E protein.

Not done in this study but we agree with the reviewer that it would be of value to determine percentage of binding Abs to E, DIII. As noted in our Introduction, the DIII domain contributes to viral attachment and DIII-specific Abs are type specific and neutralizing.

3.	Since ADE is of concern particularly in areas with DENV co-circulation, it will be important to show the absence or limited generation of the cross-reactive antibodies or lack of cross-neutralization with DENV.

We agree with the reviewer that capacity of our VLP-induced nAb to enhance either subsequent ZIKV and/or DENV infections is important. Although beyond the scope of this study, we have plans to address this topic.

Reviewer #2: This study is thorough and the manuscript is well-written. Despite the decrease in ZIKV incidence the virus still represents a potential public health threat and vaccines may be needed to stem the spread of a new epidemic should it arise. The use of several methods to measure neutralizing antibodies is impressive and not typical for comparable studies. The use of both mice and primates is likewise impressive. The use of a virus now thought to harbor tissue culture adaptations that result in some in vivo attenuation is unfortunate (if true) but not restricted to this study. However, this information should be included for context as the field moves forward with vaccines and therapeutics.

(Line **651** Tracked changes document)

Yes, we agree and the following language has been added to the Discussion (Line **651** Tracked changes), “It should be noted that low titer passage of PRVABC59 has been shown to acquire mutations in E (V333L) and NS1 (W98G) which attenuates pathogenesis in mice [Duggal Virol 2019]. It is not known how these mutations affect viral pathogenesis in NHPs. The virus was sequenced prior to the NHP challenge whereby the consensus sequence (more than 50% of the sequences assessed) did not have the E (V333L) mutation but did have the NS1 (W98G) mutation in approximately 62% of the sequences tested. This mutation may or may not be a contributing factor in low level of viremia detected in the NHPs.

Reviewer #3: see attached document

--------------------

PLOS authors have the option to publish the peer review history of their article (<a href="<https://journals.plos.org/plosntds/s/editorial-and-peer-review-process#loc-peer-review-history>" target="_blank">what does this mean?</a>). If published, this will include your full peer review and any attached files.<br><br>

If you choose “no”, your identity will remain anonymous but your review may still be made public.<br><br>

<b>Do you want your identity to be public for this peer review?</b> For information about this choice, including consent withdrawal, please see our <a href="<https://www.plos.org/privacy-policy>" target="_blank">Privacy Policy</a>.

Reviewer #1: No

Reviewer #2: No

Reviewer #3: No

**PNTD-D-20-01817:**

**Zika virus-like particle vaccine protects AG129 mice and rhesus macaques against Zika virus**

We would like to thank the reviewers for their careful reading of the manuscript and providing comments and suggesting edits for improving the quality of the manuscript.

Authors have tested immunogenicity and efficacy of a previously developed ZIKV prM/E VLP vaccine in A129 mice and NHP primates. Authors claim that co-administration of alum as an adjuvant with their VLP vaccine is dose-sparing. The manuscript could benefit from more proofreading (for grammatical mistakes and English editing) and to improve the flow. It was very hard to follow the results section, as data was not written properly in the text nor were the methods.

We have addressed the Materials/methods, Results, and Discussion sections with attention to accuracy and flow as noted below in specific comments.

In the introduction it would be important to mention other ZIKV vaccines that are in development especially other VLP vaccines and explain the novelty of their approach. A link to and more background on their previous work (study by Espinosa et al 2018; https://www.sciencedirect.com/science/article/pii/S2352396417304917#s0045 ) needs to be provided. This will help explain the background of this VLP candidate and point out the novelty and significance of the current (presented) work. It is also important the authors point out that this VLP is a chimera of 2 ZIKV strains. It would be appreciated if the authors can specify that in the introduction and also specify the details of the ZIKV cassette plasmid in methods (line 117).

(Line **75** Tracked changes document)

This topic is partially addressed in the discussion, we agree with the reviewer that this information should be expanded and also included in the Introduction section. The following language has been added to the Introduction to inform the reader of other ZIKV vaccines in development, especially other VLP vaccines. This additional language also addresses the novelty of our vaccine of using a chimeric approach and links it back to information provided in our previous publication. The following specific language has been added to the introduction (Line **75** tracked changes), “Various traditional vaccine platforms used in ZIKA vaccine development include DNA, viral-vectored (Measles/Adenovirus/VSV/DENV/MVA) and inactivated virions. The mRNA vaccine approach although ‘not traditional’ has recently been developed to address COVID-19 disease and has been granted Emergency Use Authorization (Pfizer/BioNTech and Moderna, December 2020). The development of VLPs, based on the use of structural proteins as a vaccine, can be achieved by different approaches including DNA (e.g., NIAID/VRC), mRNA (e.g., Moderna), and viral vectors (e.g., Themis Bioscience). We choose to generate the VLPs for use as a vaccine by *in vitro* introduction of plasmid DNA encoding Zika structural protein (prM-E) genes into mammalian cells. We used a chimeric vaccine design whereby the E ectodomain was derived from a recent circulating Brazilian strain SPH2015 and the prM/E stem-anchor sequences were derived from the African MR766 strain. The strategy for the chimeric design was two-fold; promote generation of an immune response to a recently circulating strain and selection of the optimal chimeric design to achieve highest VLP yield *in vitro*. As described in our previous publication [36], we demonstrated that mice immunized with ZIKV VLPs induced immune sera that upon transfer to AG129 mice were protected against ZIKV challenge. Studies described herein continue development of our VLP vaccine development approach in AG129 mice and rhesus macaque animal models.”

(Line **133** Tracked changes document)

The following language has been added to the Materials and Methods section to specify the details of the ZIKV cassette (Line **133** tracked changes), “The ZIKV cassette used to generate the VLP consists of: 1) the human IL2 signal sequence (MYRMQLLSCIALSLALVTNS) for prM; 2) the prM sequence contains 168 aa, starting with AEI and ending with AYS from the African MR766 strain; 3) the E ectodomain from the Brazilian SPH2015 strain contains 405 aa, starting with IRC and ending with SGS; and 4) the stem-anchor from the African MR766 strain contains 99 aa starting with TIG and ending with VSA.”

It is necessary that the authors explain the novelty of their vaccine candidate – ie how does it compare to other ZIKV VLP vaccines and envelope-based vaccines? Discussion needs more detail (depth), not just sweeping statements on 3 vaccine candidates and alum as an adjuvant.

(Starting Line **668** and **676** Tracked changes document)

The discussion has been expanded to provide more detail on the 3 vaccine candidates. Language has been added in the Discussion to specifically address differences in VLP approaches compared to our development strategy. The Discussion text now reads, “Other groups have utilized the prM-E structural genes as a ZIKV vaccine approach using delivery formats other than our VLP candidate vaccine, e.g: DNA ([29]; Ph1 NCT02809443; Ph1 NCT02887482; Ph2 NCT02996461); RNA ([72]; Ph1 NCT03014089), inactivated virion ([33]; NCT02963909; NCT02952833; NCTO2937233; CTRI/2017/05/008539; NCT03343626; NCT03425149); or viral-vectored [35], e.g., dengue, vaccinia, adenovirus, and measles (Ph1 NCT02996890). Differences in VLP composition were made by addition of the capsid protein to the prM-E structural proteins [73, 74]. Garg and colleagues provided data suggesting that superior nAb titers were induced by VLPs expressing the capsid protein compared to VLPs expressing only prM-E, and this effect was observed at the lowest dose tested, 3µg. Additional animal studies and even immunogenicity comparisons in humans would likely be required to confirm that capsid inclusion in the VLP structure results in a more potent immunogen. In summary, differences in VLP vaccine development are based on delivery platform utilized (encoded in nucleic acid or delivered as a particle), composition of VLP (prM-E +/- capsid structural proteins), and ZIKV strain sequences used in VLP composition. Of note, the following are examples of ZIKV strains used in VLP composition; 1) the NIAID/VRC DNA vaccine encodes sequences from the French Polynesian isolate strain H/PF/2013, [69]; the Moderna mRNA vaccine encodes sequences from the Asian ZIKV strain Micronesia 2007 [72], and as noted previously, our VLP E ectodomain is derived from the Brazilian strain SPH2015. The rationale for strain selection, in part, is based on belief that immune responses induced specific for these ZIKV structural genes will be protective against current circulating strains.

Data on the VLP production and purification (Figure 1) is not novel, as it was reported in the authors’ previous publication by Espinosa et al 2018. Also, as there is no mention of E stem anchor in the plasmid design (line 117- VLP design, purification and characterisation) nor is it indicated on the plasmid schematic- please correct this and explain. Maybe this Figure should be a Supplementary figure outlining vaccine design and manufacture as it is a repetition of what has been previously published (the wording in Fig legend is exactly the same as what has been published previously in Espinosa et al 2018).

Due to confusion as the reviewer noted, we believe it is important to keep Fig 1 but clarify and include more detail in vaccine construct design (See new Fig 1A and Fig 1A legend), i.e., signal sequence for prM and stem-anchor region of E are now clearly indicated in the diagram.

Line 117 (Line **133** in Tracked changes document)

Information regarding stem anchor is now provided in both the Fig 1A diagram and in Materials and Methods section (Starting Line **133** tracked changes) The ZIKV cassette used to generate the VLP consists of: 1) the human IL2 signal sequence (MYRMQLLSCIALSLALVTNS) for prM; 2) the prM sequence contains 168 aa, starting with AEI and ending with AYS from the African MR766 strain; 3) the E ectodomain from the Brazilian SPH2015 strain contains 405 aa, starting with IRC and ending with SGS; and **4) the stem-anchor from the African MR766 strain contains 99 aa starting with TIG and ending with VSA**.”

Also, it would be nice to have the VLP formation confirmed by TEM. The authors do not necessarily need to do this for this publication as they have shown previously that VLPs are made- however it would be good if they can provide assurance that they perform batch testing of their VLP preparations.

(Line **154** in Tracked changes document)

We have added the following text to inform the reader regarding VLP characterization, “Each batch of VLP is quality tested to confirm identity and purity by performing SDS page, Western blot, and dynamic light scattering (DLS) analyses.”

**Immunogenicity in AG129 mice**

Correct the subheading line 360 (Line **391** in Tracked changes document)- it is AG1**2**9 mice not AG19. Link data that is being discussed to figures at end of sentences.

This error has now been corrected (Line **391** in tracked changes) to read, AG129. The mouse and rhesus immunogenicity/efficacy Results section has been reviewed and special attention was given to link data being discussed to Figs at end of sentences.

Data in Figure 2B is not discussed, there needs to be a better link between nAb levels after prime and after boost (Fig2C). Has statistics been done to compare the different doses, as most of the reported statistics are when compared to alum only group? Please specify if so. You state that all but some vaccinated groups have significant levels of nAb when compared to control, but you have obviously done a titration study to determine the optimal and/or minimal dose required for protection. As such you need to show if there are any statistical differences between different doses.

As noted previously, please see page 2 for details indicating that statistical analyses were performed for **all** pairwise comparisons noted in Tracked Changes document, e.g., Lines 349, 418,459, 473,543, 545, and 583.

It should be noted that Fig 2B and 2C results/links are discussed in the following text, (Starting **Line 398** Tracked Changes document), “Significant nAb responses, as measured by the RLucNT_50_ assay, were evident for the 10µg VLP +/- alum and 1µg VLP + alum dose groups relative to the alum only group following the prime immunization (Fig 2B). The prime responses were increased from 10 to 80-fold following the second, booster immunization (Fig 2C). The increase was most evident at the lowest dose of 0.1µg formulated with alum where the mean Ab titer was boosted approximately 80-fold, 59 to 4,968. Vaccination, in the absence of adjuvant, following a booster immunization was dose-responsive, with geometric mean (geomean) nAb titers of 384 (0.1µg), 1,065 (1.0µg), and 8,670 (10µg). VLP vaccine formulated with alum adjuvant resulted in consistent production of geomean nAb titers in the range of 4,000-6,000 regardless of dose, Fig 2C.”

What was the correct challenge dose? In methods (line 157) authors specify that mice were challenged with 100-200 PFU of PRVABC59 but in the figure legend of figure 3 they say ~100CCID50 (line 400)?

This error has been corrected. In both Methods section (Line **178** Tracked Changes document) and Fig 3 (Line **426** Tracked Changes document), the correct challenge dose now reads ~100CCID_50_.

**NHP immunogenicity and efficacy**

How did the authors predetermine a 15:1 alum:VLP ratio for NHP immunisation dose?

“We titrated both the VLP and alum doses in the NHP studies in contrast to the mouse studies that held the alum dose constant for each of the VLP titration doses. This was done to increase the likelihood of observing a vaccine ‘dose-effect’ on immunogenicity and efficacy.” This language is now included in the Materials and Methods section (Line **230** Tracked Changes document) to inform the reader why this dosing strategy was performed.

Fig 6A- I am confused by multiple blood draws after challenge- there appear to have been 8 blood draws in a time period of 10 days

This information is correct and no changes were made to the manuscript. The multiple blood draws were used to evaluate RNAemia. As stated in the Materials and Methods section (Starting Line **328** Tracked Changes document), “RNA was isolated from serum samples collected on Study Days 0, 56, 58, 59, 60, 61, 62, 63, 66, and 70/71 (which are days -56, 0, 2,-7, 10, and 14 or 15 post-challenge) using the cador Pathogen 96 QIAcube Kit (Qiagen, Strasse 1 40724 Hilden, Germany) on the QIAcube HT instrument (Qiagen) and stored at ≤-70°C until RT-qPCR analysis.”

Line 465 (Line **513** Tracked Changes document) (- specify when the blood samples drawn

The days blood samples were obtained are now indicated. The text now reads (Line **513** Tracked Changes document), “Blood samples were drawn following each VLP immunization (Days 28 and 56), and viral challenge (Days 56, 58-63, 66, and 70/71 of the study which are days 0, 2-7, 10, and 14/15 post-challenge), Fig 6A.”

Line 467 (Line **515** Tracked Changes document): on which days was humoral immunity evaluated? If at every blood draw, say so?

The time points for which humoral immunity was assessed are now indicated. The text now reads (Line **515** tracked changes), “ZIKV VLP-induced humoral immunity was evaluated on Days 28, 56, and 70/71 by virus-specific nAb assays: PRNT, MN, and RlucNT.

Line 468 (Line **518** Tracked Changes document): on what days post challenge was VL quantified?

The Days VL were quantified post-challenge are now indicated. The text now reads (Line **518** Tracked Changes document), “Protection following viral challenge was evaluated by viremia (PFU/mL) and viral RNA genome equivalents (GE/mL) observed in the plasma on Days 56, 58-63, 66, and 70/71 which are days 0, 2-7, 10, and 14/15 post-challenge.”

Figure 6 (Line **527** Tracked Changes document): figure legend. I am completely confused by animal numbers? Here you say 6 NHP per group, in methods you say 5 per group with an extra one added randomly. Where? Your graphs clearly show data for only 5 animals per vaccine dose? Please be specific for each group and correct text accordingly.

The error has been corrected to indicate 5 NHPs per group in Fig 6. The text now reads (Line **527** tracked changes, “Rhesus macaques (**5** per group) were immunized on the indicated days with	“

Again, please if you are doing a dose titration study you must specify results obtained for each. You also must compare different doses and note if significant or not (for nAb data).

Please note this was addressed previously page 2 but added again specifically for NHP.

In regard to statistical analysis of different sample dose groups, these were generally not significant. However, outcomes of these analyses are now included in the text. Results have been revised to indicate the absence of significant differences between VLP groups for every statistical test performed.

Different VLP dose induction of nAb responses have been compared for statistical differences and added to the text, e.g., (Starting Line **539** Tracked Changes document) “By Day 28, following the primary immunization, only the high dose group of ZIKV VLP 20µg induced significant nAb responses compared to the alum only group (300µg) as evaluated by PRNT_50_, MN_50_, and RLucNT_50_, with geomean titers for the 20µg group being 38, 63, and 153, respectively. On Day 28, the 20µg VLP dose induced nAb titers that were also significantly higher than those induced by the 1.25µg dose. Following the booster immunization (Day 56), both the VLP 20 and 5µg doses induced significant nAb responses compared to the alum only group. **There were no significant differences in nAb responses between the different VLP dose groups**.”

Authors also show nAb titers 15 days post-challenge (day 70/71) but do not mention them in results section. Please correct this. Either remove them from the graphs or report the data.

(Starting Line **548** Tracked Changes document)

The (Day 70/71) nAb data are now indicated in the text. The following language was added (Starting Line **548** Tracked Changes document), “At study end, Day 70/71, geomean nAb responses were elevated compared to the Day 56 viral challenge time point, Figs 6B, 6C, 6D. At this final time point there were no significant differences in responses between VLP doses or between doses and the alum only group. The end-of-study responses, in part, likely indicate de novo synthesis of nAb production based on viral replication.”

For the NHP model, it would be helpful to say what viremia looks like when NHPs are challenged with this dose, i.e. when does it peak and when is it cleared?

(Starting Line **574** Tracked Changes document)

The following language was added to the Results section to address peak virema and clearance. “The ability of the ZIKV VLP vaccine to prevent viremia as monitored by a plaque assay was evaluated on Days 0, 2-7, 10, and 14/15 post-challenge (Fig 8). **Peak viremia** occurred on days 2 and 3 post-challenge and **no viral plaques were observed by 5 days post-challenge** (Fig 8A). Only 3 control animals in the alum only group and 1 animal in the low dose vaccine group (VLP 1.25µg) were positive for viremia in at least 1 plaque assay. Specifically, the following NHPs in the alum only group were observed with viremia: NHP #RA3056 (133 plaques on day 3 post-challenge); NHP #RA3111 (133 and 100 plaques on days 2 and 3 post-challenge, respectively); and #RA3281 (944 and 278 plaques on Days 2 and 3 post-challenge, respectively). NHP #RA2555 in the VLP 1.25µg + alum dose group demonstrated viremia, i.e., 100 viral plaques on day 4 post-challenge. **No statistically significant differences in viral load on any day were observed (Kruskal-Wallis plus all possible Dunn’s pairwise comparison tests)**.”

Line 491 - if 20ug dose was not significantly greater than 5ug dose, specify. Also specify if 5ug was greater than 1.25ug dose?

(Starting Line **542** Tracked Changes document)

We have now added the following language to address significant differences between doses, “On Day 28, the 20µg VLP dose induced nAb titers that were also significantly higher than those induced by the 1.25µg dose. **There were no statistically differences between other pairwise VLP dose comparisons**. Following the booster immunization (Day 56), both the VLP 20 and 5µg doses induced significant nAb responses compared to the alum only group. **However, there were no significant differences in nAb responses between the different VLP dose groups**.”

Line 577 (Starting Line **648** Tracked Changes document): authors mention that a challenge dose may account for the failure to detect infection in alum only control NHP. I disagree with this statement as many different studies have shown that rhesus macaques can be infected with lower doses than what the authors have used in their study (some have even used 10^3^ PFU (Abbink et al 2017, Sc Transl Med)).

Yes, rhesus macaques can be infected with lower doses but only using RT-qPCR as a readout.

We believe the Abbink et al 2017 reference indicates ‘infection’ using viral copies/mL as detected by PCR and not viremia, i.e., measure of live virus. We indicated the readout as viremia, i.e., plaque assay. Additionally, Abbink challenges with a different ZIKV strain, i.e., Brazil ZKV2015. They specifically state that RT-PCR assays were used to monitor viral loads. Viral loads were confirmed by PFU assays **but no PFU data were provided to ascertain level of viremia obtained following challenge**. We still believe that a higher viral challenge dose of the specific stock used in our studies may have afforded higher detection of viremia since only 3 of 5 control macaques were positive by the viral plaque assay. Another reviewer also addressed this topic and we have now included the Duggal ref (starting Line **653** Tracked Changes document) which notes that there may be attenuating mutations in PRVABC59 that has been passaged at low MOI.

Line 582 (Please note Line 663 and 671 in Tracked Changes document): There are many more vaccine candidates that have been developed using ZIKV structural genes, not just the 3 that the authors mention. They should be mentioned (vectored, inactivated ZIKV….).

(Please note Line **668** and **676** in Tracked Changes document)

Additional vaccine candidates, inactivated and viral-vectored have been included in the text. “Other groups have utilized the prM-E structural genes as a ZIKV vaccine approach using delivery formats other than our VLP candidate vaccine: DNA ([29]; Ph1 NCT02809443; Ph1 NCT02887482; Ph2 NCT02996461); RNA ([68]; Ph1 NCT03014089), **inactivated ([ 33]; NCT02963909; NCT02952833; NCTO2937233; CTRI/2017/05/008539; NCT03343626; NCT03425149); or viral-vectored [35], i.e., dengue, vaccinia, adenovirus and measles (Ph1 NCT02996890)**.” ..and “**In summary**, differences in VLP vaccine development are based on delivery platform utilized (encoded in nucleic acid or delivered as a particle), composition of VLP (prM-E +/- capsid structural proteins), and ZIKV strain sequences used in VLP composition. Of note, the following are examples of ZIKV strains used in VLP composition; 1) the NIAID/VRC DNA vaccine encodes sequences from the French Polynesian isolate strain H/PF/2013, [69]; the Moderna mRNA vaccine encodes sequences from the Asian ZIKV strain Micronesia 2007 [72], and as noted previously, our VLP E ectodomain is derived from the Brazilian strain SPH2015. The rationale for strain selection, in part, is based on belief that immune responses induced specific for these ZIKV structural genes will be protective against current circulating strains.”

Minor comments:

Line 42: As ZIKV infection has been causally linked to CZS, reword the sentence (remove word potentially).

(Line **42** tracked changes) We have removed the word “potentially” which now indicates a more definitive link between infection and fetus harm.

Line 123 (Line **142** Tracked Changes document): Please provide details on VLP purification or if the same as in Espinosa et al 2018 link to the reference.

VLP purification was the same as Espinosa and was therefore linked to the ref (Line **142** Tracked Changes document, “For VLP purification, the clarified harvest was concentrated and diafiltered into load buffer and purified using a two-column chromatography process as described previously [36].”

Line 186 (Line **210** Tracked Changes document)-Please specify what the vaccine dose/alum groups were and how many animals per group? i.e. 20ugVLP/300 ug alum – (2 males, 3 females) and so on.

The requested male/female allocation information has been added to text, “Specifically, males and females were allocated as follows: alum-only control group (3M,2F); 20µg VLP/300 µg alum (2M,3F); 5µg VLP/75µg alum (3M,2F); 1.25µg VLP/18.8 µg alum (2M,3F).”

Line 364 (Line **396** Tracked Changes document) for clarity can you please specify the challenge dose and route

The text has been changed to add the information and now reads, “Groups of 10 adult male and female AG129 mice were vaccinated with ZIKV VLP following a prime/boost schedule prior to challenge on Day 44 with a Puerto Rican isolate of ZIKV, PRVABC59 at **a dose of ~100 CCID_50_ delivered SC injected into the inguinal space between the back-right leg and the body in 0.1 mL volume**.”

Line 366 (Line **400** Tracked Changes document) “induced upon a prime “ remove “a”

The “a” has been removed.

Line 367 (Line **401** Tracked Changes document) “following the a second” remove “a”

The “a” has been removed.

Line 399 (Line **443** Tracked Changes document): correct Study day 44

Language was changed to “on day 44 of the study…….

Line 387 (Line **424** Tracked Changes document) Please specify vaccine doses and also specify the challenge dose and route.

The specific vaccine and challenge virus doses and routes have been added. The text now reads, “Mice were immunized with a dose titration (10, 1.0, 0.1µg VLP each dose formulated in 100µg alum) of ZIKV VLP (50µL each hind limb, 100µL total) by the IM route. Mice were challenged on Day 44 with ZIKV strain (PRVABC59) with ~100 CCID_50_ delivered SC injected into the inguinal space between the back-right leg and the body in 0.1 mL volume.”

Line 465 (**Line 511** Tracked Changes document): correct PR**V**ABC59 and specify dose and route of challenge

We have made the correction and it now reads, “…..followed by a ZIKV PRVABC59 challenge on Day 56. Challenge virus was administered SC in the center of the back just caudal to the scapular region with a 1.0mL dose containing 4.74 × 10^4^ PFU virus.”

Line 540 (Line **610** Tracked Changes document): please specify statistical test that was used

The text now reads, “Statistical differences between groups (by Kruskal-Wallis analysis plus Dunn’s multiple comparison tests for all pairwise comparisons) are indicated in the text.”

Statistical tests used are now indicated Line **610**
